# Supplementary material for: The “Walking Estimated Limitation Stated by History” (WELSH) visual tool is applicable and accurate to determine walking capacity, even in people with low literacy level
Source: PLoS One. 2022 Jan 13;17(1):e0260875. doi: 10.1371/journal.pone.0260875 (PMC8757989; doi:10.1371/journal.pone.0260875)
Supplement: Attachment — Submitted filename: ANSWERS.docx [file pone.0260875.s002.docx]

**Answers to reviewer #1:** This is an interesting study describing the feasibility of WELSH questionnaire in subjects with different literacy levels. It is also reported the correlations between WELSH score and 6-minute walk test performance. The topic is remarkably interesting bringing a solution to assessment of walking capacity by questionnaire in illiterate persons, which is common in several countries.

Thank you for this excellent summary of our work.

Comments:
1) I really did not understand the questionnaire. I did not understand how the clock is used in this estimative. Please, provide sufficient details to understand the instrument.
We thank the reviewer for this comment and have tried to complete the revised version according to this suggestion. We apologize that the first version of the manuscript was probably not providing sufficient details to allow an understanding of the WELSH concept. We had the questionnaire already published but do understand that a better explanation was required to help the readers. The participants just had to put a mark on the required duration on the clock. We hope that the manuscript is now easier to read and are ready to complete the text further if necessary.

2) In same way, what are the instructions provided to the participants. Please, describe in detail what was sad in first and second rounds.
We thank the reviewer for this comment and have tried to complete the revised version according to this suggestion.

3) The use of several evaluators includes an inter-evaluator error for all assessments. It should be described. For example, the relationships were similar when the tests were applied for novice and senior students? Men and women? Etc. Given your design, this is a main point.
Thank you for this comment. The evaluations were all performed by novice students only and it was indeed one of the interests of the present study that was probably not sufficiently described or explained. We must recognize that we have not recorded the fact that the students were males or females. A paragraph and a reference have been added to the text to underline this important point. The question is clearly of interest and future studies might be done for that. Thankfully, due to the high number of evaluators adjusting for evaluators does probably make little sense (because each observer included only 3 participants) but it is a clever advice for our future protocols and if a limited number of observers include a lot of participants adjustment for observer characteristics should be needed. This has been added as an important point of discussion to the manuscript.

4) Please provide details on how the training for these students happened. It was during class? How many students per class? Did they receive a material? Did they perform some understanding test? What instructions were done regarding the recruitment? How can we ensure that the data was really collected?
Thank you for this important comment and again sorry that the initial manuscript was quite poor on this description. The latest point is of interest and explains why the student were working by groups of two to be sure that data were really collected.

5) Same points for 6MWT. Did participants perform the test in the same corridor? What instructions were done? The first stop is not a recognized marker in the test. I suggest excluding it.
Sorry that this point was unclear. No clearly not, the students did not do the tests in the same corridor… this is why they were provided a 30 m rope with cones to each group. The distance to first stop is used in the vascular literature with 6 -min walk tests but we do agree that it is not routinely used. Further removing it from the manuscript simplifies the manuscript and we thank the reviewer for this suggestion.

6) How anthropometric and demographic data were assessed? Please provide more detail on how information from table 1 were obtained.
We humbly ask the reviewer to consider the context of the study that was performed in one of the poorest countries in west Africa. It was clearly impossible to have scales for weight the anthropometric values reported are in most cases those self-reported by the patients. Note that for stature it is noted on each ID card and was retrieved from the ID. This is a real-life situation in a very low-income country. We did our best to perform the study on rigorous ethical standard and methodology, but we also had to be pragmatic on feasibility. Should this point be added to the discussion? We did not do it because we did not want to provide a miserabilist view of this work because we do think that it is a unique study, despite obvious contextual limitations.

7) The background to use 400m as a criterion is frail. I suggest converting the data as a percentage of predicted that adjust for sex, age and BMI (please see Ann Vasc Surg 2021 Jan;70:258-262). This adjusted data could be used for correlations.
Thank you for this comment used for PAD We choose 400 m because a large series of recent studies proposed the 400-m walking test as a way of defining walking limitation PMID: 34283660 PMID: 34283660, PMID: 33066134, PMID: 34283660. Further there are various equations proposed in the literature to estimate walking distance from Age/sex BMI, or age height weight and sex. When comparing the observed results of our group to these equations we found a mean difference with the expected results of -217 m -172m and -360m for three of the selected equation respectively (The one from Brazil being the first result). Clearly our population has lower results than expected from a general population which is logical due to the recommendation done to recruit. The second question that arises there is: what should be considered the normal or abnormal value? Indeed only few of the available equation provide their standard deviation from the mean and when they do the unsolved question is “should we consider a low value be minus 1SD or minus 1.5SD or minus 2SD. For the reviewer information the figures below represent the differences observed between measured distances and theoretical equations for 3 equations proposed in the literature (among which the one that you proposed is the first one), the other two are:

- Troosters T, Gosselink R, Decramer M. Six minute walking distance in healthy elderly subjects. Eur Respir J [Internet]. 1999 Aug;14(2):270–4. Available from: http://erj.ersjournals.com/content/14/2/270
- Nusdwinuringtyas N, Widjajalaksmi, Yunus F, Alwi I. Reference equation for prediction of a total distance during six-minute walk test using Indonesian anthropometrics. Acta Med Indones [Internet]. 2014 Apr;46(2):90–6.

As shown the average distance that was obtained was lower than the average estimated MWD but clearly depended on which equation was used. Following the reviewer suggestion, we have removed reference to 400 m as the distance to discriminate the presence from the absence of limitation.

We propose below just for the reviewer information an analysis based on the correlation of the WELCH score against the difference from theoretical value based on the three equations that were used for the above analysis.

Results were r=0.402 (p=0.001) with “difference from theoretical MWD” = 2.72*WELCH-337

Results were r=0.576 (p=0.001) with “difference from theoretical MWD” = 3.97*WELCH-383

Results were r=0.170 (p=0.001) with “difference from theoretical MWD” = 1.02*WELCH-414

None of these do better that the use of MWD not adjusted for theoretical values and the problem there would be to justify the used of one of the models rather than another.

8) A table with the data regarding the correct filling by literacy level analyzing with a chi-square test would be useful.
The data are provided page 9 line 180-194. Since we added a table to the manuscript we have left the results as a text.

9) I strongly suggest removing the suggested equation from the manuscript. Neither the design nor the statistical methods were robust enough for it.
We have removed this from the conclusion as suggested

10) Figure 7 must be improved. The use of 3D strategy difficult the interpretation. The vertical axes do not have title. It is also not clear what is the difference between the left and right figures. This data could be presented in a table.
We have changed table 7 to account for the present suggestion.

11) It is not clear why higher correlation coefficients were observed in illiterate participants. I expected at least similar results between literate and illiterate, as literate persons are also able to understand the images.
We completely agree with the reviewer and have no satisfactory explanation for this. Could it be that subjects with high level of literacy were less careful about the filling considering that this was a too simple tool for their level of education? Could it be that the range of observed value was lower in educated patients (Which almost automatically trends to reduce the quality of the correlation)? Could it only be by chance?

12) I suggest attenuating the statement of validity of questionnaire in the conclusion. The methods are not robust enough for it.

We have smoothed our conclusion to account for this suggestion.

**Answers to reviewer #2**: This paper purports to contribute data supporting the use of a visual tool that will enable individuals to self identify whether they have a walking limitation. It evaluates whether the tool is appropriate for individuals with widely varying levels of literacy. However, the methods and descriptions of the project do not not technically sound and are not well presented. Little justification is provided on how the tool should be used in clinical or research settings, and modest correlations (if the purpose is to demonstrate "Applicable and accurate walking capacity") are reported as constituting sufficient evidence.

Thank you for this summary of our work. We agree that the present study is only one step forward in the validation of the WELSH tool, that will need external validation, analysis of sensitivity to changes of reliability of internal consistency, Evaluation of the presence of potential ceiling effect, etc… Concerning the modest correlation we wish to underline that the present tool is clearly in the same range as most of other available tools (including the WIQ). We apologize that the description of how the tool was used in the present study (and could be used by others) was missing. This has also been underlined by reviewer 1 and we have completed the manuscript to account for these perfectly justified criticisms.

The WELSH tool
The tool itself is described in a very confusing manner. The authors report that another paper demonstrates the validity of the approach, but there is no summary of validation data, nor a listing of how the tool can be used to benefit patients or society.

This comes down to the previous comment and the manuscript has been completed to provide more information on this important point.

The tool employs a scaling strategy that seems ad hoc and makeshift. Patients make a mark of a clock to identify the perceived maximum walking time for three different walking speeds, and then made self-rating of their own pace. Oddly, if the rating was less than 20 minutes, 1 point was added for each 5 minutes of rating If it were more than 20 minutes 1 point was assigned for each 10 minutes. This seems arbitrary, and I wondered why some logarythmic or other strategy might have provided a more comprehensible scale. The results placed each individual on an 8-point scale, then utilized as an equal interval scale and summed across the three different speeds. Then (also odd in my view) this sum was weighted (multiplied) by the self-rating of walking capacity (a 4 points scale). This final score was taken to be a measure of walking capacity. (I confess that I am not familiar with previous strategies of self-reported physical capacities, then the current strategy may be widely employed by clinicians. However, the statistical treatments of these unusual scale values (as presented) is not warranted.

We thank the reviewer for this comment that requires some extensive explanation. Initially the first tool that was developed was the EACH-Q questionnaire that was built on the concept that patients have reduced capacity when walking faster and that patients may apparently report satisfactory walking capacity if their usual walking speed is slow. Later, the EACH-Q has been simplified and slightly changed to the WELCH questionnaire with the idea of making a toll that would result in a score ranging 0 (inability to walk) to 100 (no limitation). The durations proposed for each walking pace followed an exponential increase. The sum of durations (scored 0 to 7) resulted in values ranging 0 to 21 and we proposed 5 different usual walking speeds… then we arbitrarily subtracted one to the sum before multiplying by 1 to 5.

In the process of transferring the concept from the textual WELCH into a visual WELSH, we faced a series of issues. First, it was clearly not easy to propose a logarithmic scale on the watch and we arbitrarily defined simple intervals that could easily be memorized by the interviewers but kept the concept that the highest duration (= or > 20 min) would represent intervals larger than short durations. Second it was relatively uneasy to represent 5 different usual walking speed and then we reduced the proposed answer to usual paces to 4 possibilities…. As a result, as the goal was to have a final score ranging 0 to 100 we had to have the first three question to have individual score that allowed a sum of 25. All these assumptions were totally arbitrary although defined on physiological concepts and it is more than likely that adjusting the coefficients proposed for the various time answers would improve the correlation with measured distance. This is indeed something that could be done from the recorded data, with for example a multilinear regression analysis. The question here is the routine use of the tool. Is gaining a few units of correlation of WELSH with 6MWT distance by applying coefficients to each answer (e.g; 2.5*the duration at low speed + 5.3 *the duration found at medium speed + 6.24 * the duration at high speed), or defining intervals of time that might represent a semi-logarithmic increase (e.g.: 2 minutes, 5 minutes, 11 minutes, 23 minutes etc… ) worth it? It would clearly result in less facility to use the tool in a routine. We completely agree that it might be of interest to confront the results of the completely arbitrarily defined score to mathematically defined coefficients. Our purpose was not there. What we only wanted to do is test the large-scale applicability to use the WELSH (define as a very simple tool both to fill and to score) and the ability of the WELSH to provide reasonable results in illiterate patients. On the scoring point of view, the fact that less than 1% of the scores calculated by student were wrong is an essential point. We doubt that a comparable result could be reached with more complex scoring rules (based on adjusted coefficients or adjusted time intervals). Please consider that the WELSH aims to be a pragmatic easy tool conceived for a pragmatic application in low-income country context with low literacy level. Clearly a more statistical approach would probably lead to improved correlation but would to our opinion lead to a decreased applicability while a more clinical and pragmatic approach is indeed very likely decreasing the quality of the correlation.

Statistical Analysis
The paper reports P values for gender differences in Table 1 which contains a mx of categorical and continuously scaled variables. Nowhere is the statistical test described (assuming the authors used t-tests and chi-squares, but there is no presentation of the test statistics. It is impossible to tell, in many instances, the precise statistical tests that were run and what the results tell us. Also, the mean scores in some comparisons are presented with one decimal place, and no decimal places in others. It was not possible to make much sense out of sentences like, "We aimed to be able to analyze data according to gender and four different literacy subgroups (8 possible subgroups) to validate our main hypothesis of a correlation 0.40 with alpha = 5% and beta.... " This suggests that .4 is the level of correlation the authors felt necessary to demonstrate an acceptable level and the required Ns are very small compared to the total N comprising the study. (The low p-values -- all <.001-- suggests an over-powered study rather than a strong effect). The authors use P-values as representations of the strength of effects of different subgroups.

On the one hand, we apologize that the statistical tests were not described and confirm that the assumption made by the reviewer are correct (Use of Chi² and t tests). On the other hand, it may have been unclear that what we dreamed of were coefficients of 0.5 to 0.6 (which is the range of coefficients observed between self-reported capacity tools and various methods of objective measurements) but we also wanted to be able to conclude that even coefficients of at least 0.40 would be statistically significant. Thereby, the calculation was based on the worth possible scenario (i.e. the worth coefficient in the smallest sub-group). Since the recruitment was unpredictable (we did not define the subjects that had to be recruited by each interviewer), The total number of subjects had to be high. This was also an interesting point to confirm that the tool was easy to use and score. Last, Yes a coefficient of 0.40 is already an acceptable level of correlation in perspective of previous results of the literature.

Regressions seem to be presented in the discussion of figure 6, but the statistics are not well described. There needs more discussion of the statistical approaches here. Authors should consider fitting the regression lines to the plots in Figure 6 which would provide a better visual representation of the strength of the linear relationships. Also in there conclusion to the paper, the statement, "In routine practice, the MWD in meters can be roughly estimated as 4 * the WELSH score +150 meters. Why not use the actual regression result coefficients? Also, given different levels of correlation for different subgroups suggests that this prediction equation would not be valid across subgroups.

Following the reviewer comment a regression line has been added to the plot. Please consider that the upper figure has been removed following reviewer one comments. As suggested by the reviewer the equation has been removed.

Figure 7 seems to contain the heart of the data analysis, but its presentation is problematic. First Figure 7 is presented but not adequately described or discussed, other than the use of phrases like, "correlation was particularly low (P=0.025)". In Figure 7, there are no labels for the Y axes and no labeling indicating that the panels on the right are of percentages of participants who were able to walk more than 400 meters during the 6-minute walking test (used to validate the too, nor that the panels on left show correlation coefficients.
Finally, Figure 7 employs an unacceptable 3-D graphing strategy. It is very difficult to determine the nature of the group comparisons.
We apologize for the poor quality of figure 7 that has been removed and replaced by a table (Table 2).

In sum, while this paper asks an interesting question regarding the availability of a tool appropriate for individuals with limited reading ability, its statistical treatment of the data is quite weak, the scaling strategy for the WELSH needs greater justification, and a better discussion of the legitimate uses of the tool, given the findings, and the limits should be provided. As well, the paper is difficult to follow at times, and the statistics are not presented in conventional formatting.

We hope that the answers provided to the different suggestions that were done to improve the initially submitted manuscript fulfill the reviewer expectation and are ready to further work on thee manuscript if some point require other clarifications.
