# Supplementary material for: The “Walking Estimated Limitation Stated by History” (WELSH) visual tool is applicable and accurate to determine walking capacity, even in people with low literacy level
Source: PLoS One. 2022 Jan 13;17(1):e0260875. doi: 10.1371/journal.pone.0260875 (PMC8757989; doi:10.1371/journal.pone.0260875)
Supplement: Attachment — Submitted filename: Response to Reviewers - Copy.doc [file pone.0260875.s003.doc]

**Reviewer #1:** Dear authors, thank for your reply. Most of my concerns were addressed, but I still uncomfortable with few points.

Comments:
1) Sorry to insist, but I still not understanding the instrument. “In brief, for the first three items, the maximum walking time that can be performed for each of 3 different walking speeds (illustrated by a turtle a human and a rabbit) must be reported. Walking speeds are considered relative to the people of the same age, family or friends”. Ok, but for what distance?

We are very uncomfortable with this comment because we are not sure that we understood it.

If the comment relies on the fact that we should have better asked for distance rather than time, we have already explained in the present manuscript (as we did before for the papers dealing with the WELCH) that evaluating time is easier than distance (specifically in open spaces)

If the comment relies on the fact that time for a defined speed may not relate to distance: mathematically distance is the product of time per speed. Then a linear relationship exists between the two for a defined speed whatever the speed might be.

If the comment relies to the fact that speed is to be understood relative to people of the same age (and might be slightly different for old or young people) it is perfectly on purpose to make the WELSH hopefully independent of age as was the WELCH (which is a text device based on the same concept but not the copy of the WELSH). A short advice has been added to the introduction to underline this point: “*an adapted version of the WELCH (and not a translation of the WELCH into images)”*

2) “We choose 400 m because a large series of recent studies proposed the 400-m walking test as a way of defining walking limitation PMID: 34283660 PMID: 34283660, PMID: 33066134, PMID: 34283660”. Note that out of 4 studies, three are exactly the same (Effect of whole-body resistance training at different load intensities on circulating inflammatory biomarkers, body fat, muscular strength, and physical performance in postmenopausal women) and the other one is in cancer patients (Association between Sarcopenia and Physical Function among Preoperative Lung Cancer Patients).

We apologize for the errors with the three same references. Our aim was only to underline that many authors have used the 400m limit in sarcopenia, cancer, cardiac, pulmonary epidemiology studies and suggested its use in review papers. (PMID: 20166006; PMID: 32068846; PMID: 16545950; PMID: 31987880; PMID: 27174883; PMID: 30312372, PMID: 31742368, and we could have added a lot of other references….). Apparently, we did not sufficiently underline that we had removed this 400m limit from our manuscript to avoid criticisms from people that may use the 400 m limit or that would rightly underline the issue of normalization (see comments and answers to the next point). We assume that the reviewer read the comments and answers but not the revised manuscript either in redline or clean version.

3) “None of these do better that the use of MWD not adjusted for theoretical values and the problem there would be to justify the used of one of the models rather than another.” The point is not what correlates better, but that 6MWT performance is influenced by clinical and demographic parameters. It is well stablished that women, elderly, obese and smaller subjects present lower values than men, taller, normal weight and young subjects. Therefore, 400m for an elderly woman indicates a better health condition than a 400m in a young men. The use of equation is an adjustment for these factors, which is stronger than the simple correlation. The suggestion is to use the equation in which the population profile is closer of your population (age, height, obesity prevalence, etc). A threshold of 84% is often employed, however, for your linear analysis it is not needed. I think this is an additional and not a substitutive data.

We agree to the suggestions of the reviewer about the difference that 400 m may represent pending on age, sex, etc… but we are a bit in trouble with these two comments of the reviewer because both rely on the initial version of our manuscript and not to the revision that was submitted. Indeed, all references to the 400 m have been removed from our manuscript and this point no longer applies to the revised manuscript as explained above.

**Reviewer #2:** This paper is dramatically improved. The authors have addressed most of my original comments. I commend the authors for the completeness with which they have addressed the reviewer comments.

1. I still have some concerns about the validity of the tool. To what purposes is the tool valid. Is it useful a a screening device? Do the scores suggest specific medical interventions? Although they do not use the term "diagnostic",there is an implied conclusion that the test has diagnostic value. A description of the situations in which the scale wold be useful wold be helpful.

We thank the reviewer for this question that requires diverse answers for the various points.

- The issue of purposes is very important and clearly will require future studies that were not scheduled until we had some idea of the questionnaire applicability in low literacy people. Use of the WELSH in the future will require various validations in various application domains. We are currently working on severe anemia in pregnancy in an African population as well as a project is ongoing in vascular patients. The WELSH is not licensed and future researchers are free to use and validate the tool in various populations and/or various diseases states
- The issue of screening device is one of the possible uses of a tool applicable to a population of low literacy.
- This issue of which score shall require specific medical interventions and a very important question although it is clearly impossible to answer to this question to date. This limit for intervention clearly depends on which disease is studied and in which population.
- That fact that we do not use the term "diagnostic", is clearly on purpose because to date we cannot claim that it has diagnostic value… at least to date. Further the potential application could also be for follow-up rather than diagnostic
- A description of the situations in which the scale would be useful would be helpful. This sentence is somehow a summary of the previous comments and we have added a short paragraph to the manuscript to account for these comments just before the conclusion

“*The fact that novice students performed the tests is also the reason why, for security reasons we excluded patients with severe co-morbid conditions. Future studies under medical supervision should be performed to test the use of the WELSH as a screening tool or in various medical or surgical conditions, as well as to define which score would suggest the need for specific medical or surgical intervention*.”

2. Why eliminate participants with known walking disabilities? Would it be advantageous to show that the WELSH scores on disabled individuals to demonstrate its validity in potential disability in others?

We totally agree with the reviewer that including disabled people would have increased the range of available data, but for security reasons of non-supervised out-of-the-lab tests we wanted to have no severely disabled patients. It is clear that including disabled subjects would have enlarged the range of available data which generally trends to improve correlation coefficients. Then the coefficients obtained are possibly improved in the future. Then it clearly is of interest in the future to do studies including disabled but we could not do it here. This limit has been added to the final sentence of the discussion reported above “*The fact that novice students performed the tests is also the reason why, for security reasons we excluded patients with severe co-morbid conditions”*

3. Should the picture version of the test been validated with the text version of the questionnaire administered to participants who can read? This would lend confidence that the picture version is measuring the same construct as the text version.

Thank you for this question. We apologize if the manuscript was a bit confusing but the WELSH **is not** a picture version of the WELCH. The WELSH is not aiming at copying the text version (WELCH) but it is only based on similar concepts (1/ estimation of time instead of distance, 2/ estimation of time for different speeds, 3/: estimation of usual speed). Consistently the calculation of score is not the same. Then there is no real objective reason to compare WELSH to the WELCH rather than to the WIQ or Vascu-QOL or any of the other available text questionnaires. We have added the following advice to the introduction to avoid confusion :” (*and not a translation of the WELCH into images*)”

4. What are the inter-correlations among the four items of the test, and What are the correlation of these items with the walking score singly? Does the proposed scaling result in a higher correlation than, for example, simply using question 4?

We thank the reviewer for this very interesting question that was worth a try and that we did not previously do. For the reviewer suggestion the analysis based on available data shows that individual questions do not better correlate with MWD than the score. We provide the results for the reviewer information but have not included them in the text: Correlation to MWD for 1/ Time at a low speed r= 0.458; 2/ Time at average speed r=0.482; 3/ Time at a high speed r = 0.479; 4/ Usual speed r=0.488; while the correlation with the final score was r=0.567. Nevertheless, these results have not been added to the manuscript but should be added if the reviewer thinks that they are essential to the readers.

5. I would have used a non-parametric correlation, such as Spearman, to avoid the criticism regarding the equal-interval assumptions required for a Pearson r and linear regression.
Thank you for your comment. We have therefore followed your advice for all the analyses and modified these elements in the manuscript.

6. Finally, I question the use of a self rating as this in epidemiological studies that are listed as potential uses of the scale, where any two participants may be applying different criteria to define similar scale value, leading to epidemiological findings that would be difficult to interpret.

We assume that this comes down to the question of estimating for walking speeds “relative to family friends. Contrary to being a limit, we advocate that this self-rating relative to people of the same age family and friends is a major advantage to have the tool conceptually adapted to (and hopefully independent of) age. To account for this comment, the following paragraph has been added to the manuscript “*Forth, the fact that speed is considered “relative to the people of the same age, family or friends” could be considered an issue with the idea that any two participants may be applying different criteria to define similar scale value. We advocate that, on the contrary, this is of major advantage to make the tool conceptually adapted (a hopefully relatively insensitive) to age.”*
